# Radiomics in photon-counting dedicated breast CT: potential of texture analysis for breast density classification

**DOI:** 10.1186/s41747-022-00285-x

**Published:** 2022-07-20

**Authors:** Anna Landsmann, Carlotta Ruppert, Jann Wieler, Patryk Hejduk, Alexander Ciritsis, Karol Borkowski, Moritz C. Wurnig, Cristina Rossi, Andreas Boss

**Affiliations:** 1 Institute of Diagnostic and Interventional Radiology, University Hospital Zurich, Zurich, Switzerland; 2 Institute of Computational Physics, Zurich University of Applied Sciences, Zurich, Switzerland; 3 Institute of Diagnostic Radiology, Hospital Lachen AG, Lachen, Switzerland

**Keywords:** Breast density, Breast neoplasms, Image processing (computer-assisted), Radiomics, Tomography (x-ray computed)

## Abstract

**Background:**

We investigated whether features derived from texture analysis (TA) can distinguish breast density (BD) in spiral photon-counting breast computed tomography (PC-BCT).

**Methods:**

In this retrospective single-centre study, we analysed 10,000 images from 400 PC-BCT examinations of 200 patients. Images were categorised into four-level density scale (*a*–*d*) using Breast Imaging Reporting and Data System (BI-RADS)-like criteria. After manual definition of representative regions of interest, 19 texture features (TFs) were calculated to analyse the voxel grey-level distribution in the included image area. ANOVA, cluster analysis, and multinomial logistic regression statistics were used. A human readout then was performed on a subset of 60 images to evaluate the reliability of the proposed feature set.

**Results:**

Of the 19 TFs, 4 first-order features and 7 second-order features showed significant correlation with BD and were selected for further analysis. Multinomial logistic regression revealed an overall accuracy of 80% for BD assessment. The majority of TFs systematically increased or decreased with BD. Skewness (rho -0.81), as a first-order feature, and grey-level nonuniformity (GLN, -0.59), as a second-order feature, showed the strongest correlation with BD, independently of other TFs. Mean skewness and GLN decreased linearly from density *a* to *d*. Run-length nonuniformity (RLN), as a second-order feature, showed moderate correlation with BD, but resulted in redundant being correlated with GLN. All other TFs showed only weak correlation with BD (range -0.49 to 0.49, *p* < 0.001) and were neglected.

**Conclusion:**

TA of PC-BCT images might be a useful approach to assess BD and may serve as an observer-independent tool.

## Key points

- Analysis of texture features on spiral photon-counting breast computed tomography is useful in the assessment of breast density.
- Texture analysis may provide as an observer-independent, objective tool for breast density assessment and serve as quality control tool.
- Texture analysis may complement breast cancer risk estimation, reflecting parenchymal tissue characteristics more precisely.

## Background

With an estimated 2.3 million new cases per year worldwide, breast cancer (BC) constitutes the most frequently diagnosed cancer among women [1]. In addition to non-modifiable risk factors, such as genetic predisposition, age, and hormonal influences, breast density (BD) is known to be an independent risk factor for developing BC [2]. Epidemiological studies have shown that the risk for BC in women with dense tissue may increase 2–6 times when compared to women with less dense tissue [3]. Although of high clinical importance, BD is often difficult to determine. Mammographic BD is defined as the relative amount of glandular tissue based on the mammographic appearance of parenchymal tissue on the mammogram. Besides the amount of fibroglandular tissue, parenchymal patterns appear to be indicative of the individual BC risk. The distribution of fatty, glandular and stromal breast tissue are assumed to be related to factors that are associated with the development of BC through unknown biological mechanisms [4].

The BD assessment is implemented into the Breast Imaging Reporting and Data System (BI-RADS) atlas of the American College of Radiology, classifying mammographic BD into four categories: *a*, describing almost completely fatty tissue; *b*, describing scattered fibroglandular tissue; *c*, describing heterogeneous dense tissue; and *d*, describing extremely dense tissue [5].

Besides the increased individual risk of developing BC, BD represents an important parameter influencing the diagnostic performance of screening mammography. While the sensitivity of mammograms for low-density breasts (categories *a* or *b*) is reported to be 87%, the sensitivity in dense breast tissue (categories *c* or *d*) decreases to 63% with the need of additional imaging. Fast and ubiquitously available, supplemental breast ultrasound is the modality of choice with an additional cancer yield of 2−4 cancers per 1,000 patients and therefore, recommended in many guidelines [6, 7]. Contrast-enhanced breast magnetic resonance imaging (MRI) is known to be the most sensitive imaging modality, even in detecting early stages of BC, one of its main indications being screening in high-risk patients [8]. Large patient cohorts like those investigated by the DENSE trial were able to show that supplemental breast MRI in women with negative mammograms results into about 17 additional cancers per 1,000 patients at the cost of an increased false-positive rate. However, false-positive rate strongly decreased in the follow-up round (from ~ 80 per 1,000 to ~ 26 per 1,000 women). Additionally, the interval cancer rate was significantly lower when performing additional breast MRI compared to mammography only (2.5 per 1,000 *versus* 5.0 per 1,000 women) [9–12].

With the striving field of radiomics, on which artificial intelligence can be applied, there is growing evidence regarding textural features of parenchymal density as an inherent, independent, biologic risk factor for developing BC [13]. Several recent studies already evaluated the importance of parenchymal texture analysis (TA) for BC risk assessment based on mammography screening [4, 14, 15]. It allows an objective assessment of tissue heterogeneity by evaluating the distribution and relationship of pixel- or voxel-based grey levels in the image. TA has already been successfully applied on computed tomography (CT) and MRI for the prediction of pathologic features, prognosis, and response to therapy for various body compartments and can also potentially be applied to breast CT [16–20].

Spiral breast CT using photon-counting detector technology (photon-counting breast CT, PC-BCT) offers a truly three-dimensional breast imaging modality. Although not implemented in any guidelines yet, it might provide an alternative to mammography or breast-MRI for BC screening, combining improved patient comfort, fast image acquisition, and good visibility of microcalcifications [21, 22]. Regarding lesion detection, the sensitivity of PC-BCT in dense tissue was reported to be higher compared to digital mammography at comparable radiation dose [23].

Existing studies on BD assessment using PC-BCT use the BI-RADS atlas to categorise BD levels, though the BI-RADS BD scale could not be directly applied to the three-dimensional nature of spiral PC-BCT [24]. Recently, Wieler et al. provided a new dedicated classification atlas in the assessment of BD based on lesion detectability [25]. Because PC-BCT is a new imaging modality, density assessment remains with high intra- and inter-reader variability, urging for an observer-independent and standardised classification system. Still, there is no standardised classification system, neither for mammograms, nor breast-CT, respecting parenchymal or biological patterns for accurate BC risk assessment [26].

The main purpose of this study was to evaluate the differences of radiomics features in different BD levels and to determine whether features derived from TA can be used to predict breast density in spiral PC-BCT.

## Methods

### Patient selection

**Fig. 1:**
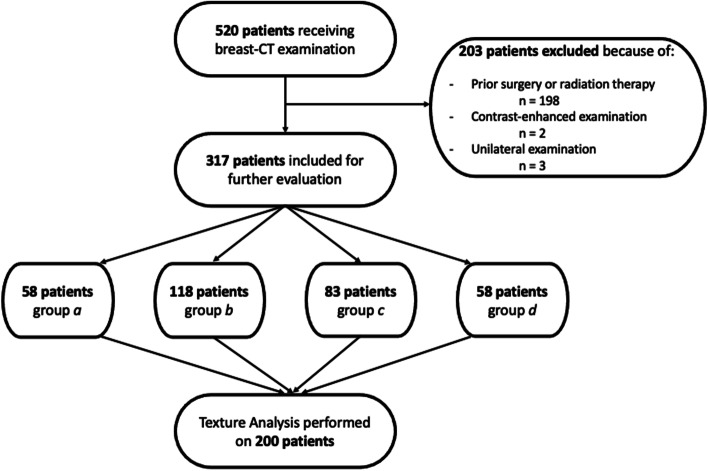
Flowchart depicting patient selection workflow

This retrospective study was approved by the local ethics committee and all patients signed informed consent for the scientific evaluation of the imaging and clinical data. All patients receiving PC-BCT examination at our institution signed informed consent about the “off-label” use of PC-BCT, including all its benefits (no compression, improved lesion detectability compared to mammography) and disadvantages (higher radiation dose, long evaluation time, not implemented in any guidelines yet) [21–23]. A retrospective search of patient data in the local database resulted in 520 patients receiving PC-BCT between January and August 2021. Patients with prior surgery or radiation therapy of the breast (*n* = 198), contrast-enhanced PC-BCT (*n* = 2) exams using intravenous injection of iodinated contrast agent, and unilateral examinations (*n* = 3) were excluded. In total, 317 patients suited inclusion criteria, resulting in 634 PC-BCT examinations, used for further evaluation. Patient selection workflow is depicted in Fig. 1. None of the patients received additional mammography prior to PC-BCT.

### PC-BCT protocol

All examinations were performed on a dedicated spiral PC-BCT unit quipped with a cadmium-telluride photon-counting detector with an area of 280 × 50 mm^2^ (nu:view, AB-CT, Advanced Breast-CT GmbH, Erlangen, Germany). The maximum diameter of the field-of-view was 190 mm and the scan length could be adjusted to the values 80, 120, and 160 mm depending on the size of the breast, resulting into 311-, 450-, or 588-slice exams, respectively. The x-ray tube exhibited a 0.3-mm focal spot size, with a 3-mm Al filtration. A fixed x-ray tube voltage of 60 kV was used, whereas the tube current could be adjusted between 5 mA and 125 mA. In all patients, a tube current of 32 mA was applied. No intravenous injection of contrast agent was applied. The examination scans were acquired in a spiral mode with the high-resolution scan protocol with scan times of 7, 9.5, and 12 s for the scan length of 80, 120, and 160 mm, respectively. Image reconstruction was done in a standard mode with a soft kernel at 300 μm^3^ voxel size with 2 × 2 pixel binning using a Feldkamp-type filtered back projection algorithm [27].

### BD classification

Breast density assessment was performed by a radiology resident with 1 year of experience in breast imaging, using raw data images in the coronal plane. According to the standard procedure of our institution, breast density in BCT was categorised in four groups using the provided classification atlas by Wieler et al. [25].

- Partial or complete involution with every lesion visible.
- Scattered glandular tissue with lesions larger than 10 mm conclusive visible.
- Heterogeneous dense glandular tissue with lesions of 10 mm potentially not visible.
- Very dense tissue with restricted visibility of lesions.

After density assessment, texture analysis (TA) was performed in 50 randomly chosen patients of each density level to provide equal distribution.

### Image selection and texture analysis

**Fig. 2:**
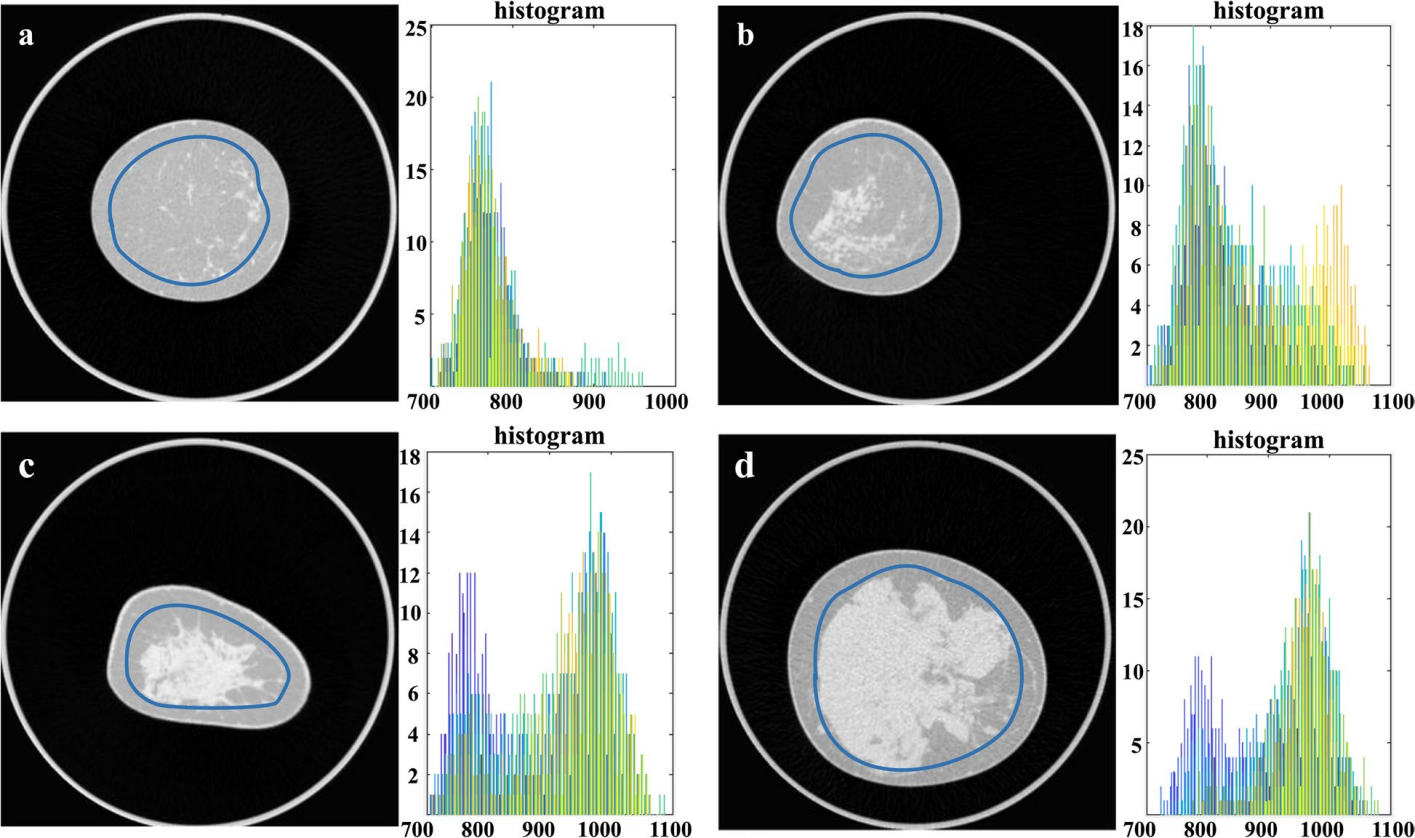
Example definitions of regions of interest for each breast density level (**a**, **b**, **c**, or **d**) and corresponding histogram graphs summarised for all 50 automatically evaluated images. Histograms depict the number of pixels found at each pixel value; whereas the left side on the *x*-axis represents lower signal values, the right side on the *x*-axis represents higher signal values. In the histograms, two peaks can be distinguished corresponding to fatty and glandular tissue, respectively. The ratio between the two peaks depends on the breast density, with class *a* mostly showing the peak corresponding to fatty tissue, whereas the histogram graph for class *d* demonstrating mostly the peak corresponding to glandular tissue

For the TA, 50 raw data images in the coronal plane of each patient were included, 25 of each side, depicting only representative slices of the breast tissue. Images showing bony structures or pectoralis muscle were excluded from the TA. In total, TA was performed on 10,000 images of 200 patients. TA was performed using an in-house developed script written in the programming language MATLAB (The Math-Works Inc., Natick, MA, USA). A region of interest (ROI) was drawn freehand on coronal images, delineating the maximum continuous area of the breast tissue by the same radiology resident. ROIs were placed with a margin of approximately 5 mm from the skin to exclude subcutaneous fat tissue from analysis, as shown in Fig. 2. Mean ROI size was not calculated. The ROI was automatically propagated to the other 49 images, both, left and right breast, and multislice texture analysis was performed. As a first step, grey-level normalisation was performed to minimise intrascanner effects. Subsequently, 19 features were computed, as listed in Table 1. The first order features (4) were directly extracted from the histogram of all grey levels in the delineated ROI. Therefore, they provided information about the signal intensity values of each pixel. The second order features (15) were derived from the respective grey-level matrices and included more information concerning grey-level distribution by accounting for the relative position of each pixel with respect of the other pixels in the image. The grey-level co-occurrence matrix (GLCM) gave information about the grey-level distribution of pixel-pairs that were separated by a given offset. The grey-level run-length matrix (GLRLM) gave information about the runs of pixels with the same grey-level values in a defined direction.

**Table 1:** Overview of texture features analysed for each image

First-order features	Second-order features	
Histogram-based	Grey-level co-occurence matrix (GLCM)	Grey-level run-length matrix (GLRM)
Variance	Contrast	Short-run emphasis (SRE)
Skewness	Correlation	Long-run emphasis (LRE)
Kurtosis	Energy	Grey-level nonuniformity (GLN)
Entropy	Homogeneity	Run-length nonuniformity (RLN)
		Run percentage (RP)
		Low grey-level run emphasis (LGRE)
		High grey-level run emphasis (HGRE)
		Short-run low-grey-level emphasis (SRLGE)
		Short-run high-grey-level emphasis (SRHGE)
		Long-run low-grey-level emphasis (LRLGE)
		Long-run high-grey-level emphasis (LRHGE)

### Human readout and reliability analysis

To evaluate the inter-reader reliability for BD assessment, a representative subset of 60 images, 15 images of each density level, was created. Images were presented in random order and BD was assessed by two expert readers with 6 years (J.W.) and 15 years (A.B.) of experience in breast imaging, in particular 4 years of experience in BCT imaging, and compared to the classification of the radiology resident.

### Statistical analysis

In a first step, a one-way analysis of variance (ANOVA) of a linear model was performed for comparison of every texture feature among different BD levels with *post hoc* Bonferroni correction for pairwise comparison between groups, using the SPSS software package (SPSS version 28.0.1.0, International Business Machines Corp., Armonk, NY, USA); only *p* values less than 0.05 were considered significant.

Confounded features identified in this step were excluded from further analysis. Further statistical analysis was performed using R (RStudio, Version 2021.09.0, Boston, MA, USA). Continuous data were expressed as mean ± standard deviation if normal distribution could be assumed or otherwise as median and interquartile range. Categorical data were given in absolute amount. After applying ANOVA, the remaining features were evaluated for correlation between the different density levels with Spearman’s rho, with values ranging from -1 to + 1, indicating whether it is a positive or negative correlation. For our purpose Spearman’s rho > 0.80, was considered strong correlation, from 0.51 to 0.80 moderate correlation, and from 0.11 to 0.50 weak correlation. In correspondence, values lower than -0.81 were considered strong negative correlation, from -0.51 to -0.80 moderate negative correlation, and from -0.11 to -0.50 weak negative correlation. Values from -0.1 to + 0.1 were considered as absent correlation. In all analyses, the threshold for assessing statistical significance was set to *p* < 0.05.

At last, a cluster analysis was performed between the BD levels to study the dependencies and associations among the features and to find the independent features. Moreover, multinomial logistic regression was performed using the R “caret” and “nnet” package (R Studio, Version 2021.09.0) to evaluate the applicability of TA for BD assessment in PC-BCT. For that purpose, data was split into a training (70%) and test (30%) dataset. Probabilities for each category (from *a* to *I*) were calculated based on the maximum likelihood estimation.

For human readout and reliability analysis, ICC for BD assessment between the radiology resident and each of the two expert readers was then calculated. According to Landis and Koch, an ICC greater than 0.80 was considered “almost perfect agreement” [28] Further, inter-reader reliabilities between the three readers were assessed by calculating κ coefficients. According to Cohen [29], κ values from 0.61 to 0.80 were considered substantial and κ values from 0.to 0.90 were considered almost perfect. In a next step, each of the three readers performed ROI placement for single-slice TA on the 60 images. Subsequently, coefficient of variation between readers was calculated for each texture feature to evaluate reliability of the proposed model.

## Results

### Patient cohort

Based on exclusion criteria, image selection resulted in 317 patients corresponding to 634 PC-BCT per breast examinations. Mean age of patient cohort was 55 years ± 9 (34–83). Of 317 patients, 58 (18.3 %) were assigned BD level *a*, 118 (37.2%) level b, 83 (26.2%) level *c*, and 58 (18.3%) level *d*. To provide equal distribution, we included 50 patients of each BD level in the final patient cohort for further TA. In total, 200 PC-BCT examinations were evaluated, each examination consisting of one image set for each side, corresponding to 400 PC-BCT examinations in total. Most women received PC-BCT for screening purpose (*n* = 239), followed by new appearing mastodynia (*n* = 49). Less common purposes for BCT examination were investigation of suspicious palpable findings (*n* = 8), follow-up examination of BI-RADS 3 or 4 findings (*n* = 6), follow-up examinations of benign lesions (*n* = 5), mastopathia (*n* = 8), secretation of the mamilla (*n* = 1), or dysesthesia of the breast (*n* = 1). In our patient cohort 135 women received additional breast ultrasound, of which 95 were referred for screening purpose.

### Texture analysis

**Fig. 3:**
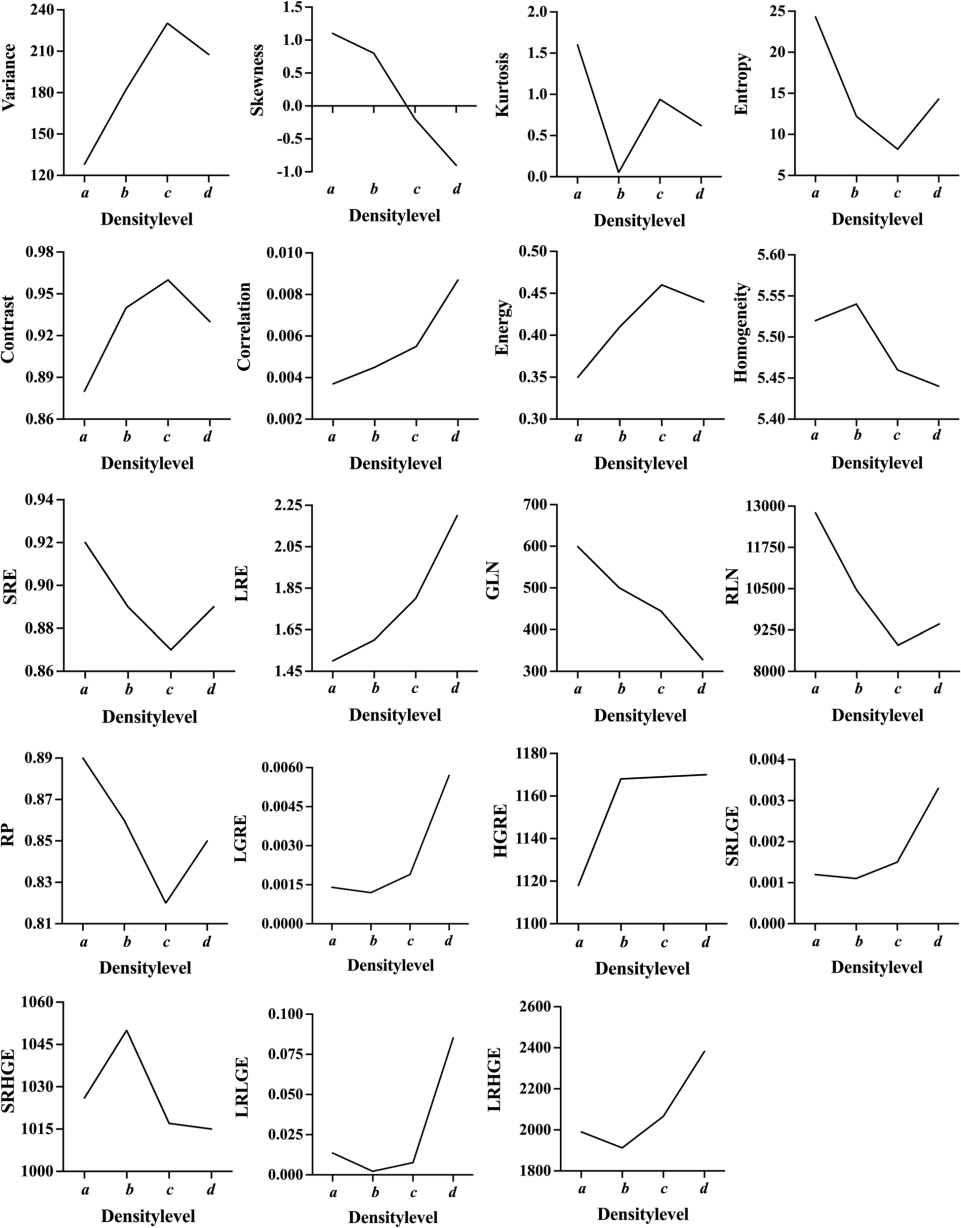
Mean values for each texture feature are dependent on different breast density levels (*a*, *b*, *c*, and *d*). *GLN* grey-level nonuniformity, *HGRE* High grey-level run emphasis, *LGRE* Low grey-level run emphasis, *LRE* Long-run emphasis, *LRHGE* Long-run high-grey-level emphasis, *LRLGE* Long-run low-grey-level emphasis, *RLN* Run-length nonuniformity, *RP* Run-percentage, *SRE* Short-run emphasis, *SRHGE* Short-run high-grey-level emphasis, *SRLGE* Short-run low-grey-level emphasis

Mean TA time for multislice datasets (50 images) for 19 TFs was 71 s. Among the different BD levels, ANOVA showed significant differences for all TFs (all *p* values < 0.001). In Fig. 3 the dependency of the median value of each TF on BD is depicted. The majority of TFs shows a systematic change for either increasing or decreasing BD. Bonferroni correction was able to exclude further TFs that showed no significant correlation BD levels. In summary, the following 11 TFs were included for further analysis: variance, skewness, kurtosis, entropy, contrast, correlation, energy, homogeneity, grey-level nonuniformity (GLN), run-length nonuniformity (RLN), and short- run low-grey-level emphasis (SRLGE). Table 2 provides an overview of TF descriptives (mean, standard deviation, 95% confidence interval) and corresponding *p* values after Bonferroni correction.

**Table 2:** Texture feature descriptives and *p* values for *post hoc* Bonferroni correction for each feature

Feature	Mean	Standard deviation	95% confidence interval		***p*** value after
			Lower	Upper	Bonferroni correction
**Variance**	187.03	74.15	185.57	188.48	< 0.001
**Skewness**	0.22	1.0	0.20	0.24	< 0.001
**Kurtosis**	0.34	1.76	0.31	0.38	< 0.001
**Entropy**	14.76	10.93	14.55	14.97	< 0.001
**Contrast**	0.93	0.05	0.93	0.93	< 0.001
**Correlation**	0.01	0.01	0.06	0.06	0.002
**Energy**	0.42	0.08	0.41	0.42	< 0.001
**Homogeneity**	16,193	0.30	5.44	5.45	0.01
**SRE**	0.89	0.05	0.89	0.89	1.0 *
**LRE**	1.77	1.66	1.74	1.81	0.1 **
**GLN**	468.09	176.12	464.64	471.54	< 0.001
**RLN**	9,432.94	4,143.13	9,351.72	9,514.16	< 0.001
**RP**	0.85	0.62	0.85	0.85	0.9 *
**LGRE**	0	0	0		0.4 **
**HGRE**	1,156.60	34.88	1,155.92	1,157.29	1.0
**SRLGE**	0	0	0		0.006
**SRHGE**	1,026.89	52.03	1,025.87	1,027.90	1.0 *
**LRLGE**	0.03	0.12	0.02	0.03	0.6
**LRHGE**	2,087.82	1,428.11	2,059.82	2,115.81	0.4

*GLN* Grey-level nonuniformity, *HGRE* High grey-level run emphasis, *LGRE* Low grey-level run emphasis, *LRE* Long-run emphasis, *LRHGE* Long-run high-grey-level emphasis, *LRLGE* Long-run low-grey-level emphasis, *RLN* Run-length nonuniformity, *RP* Run-percentage, *SRE* Short-run emphasis, *SRHGE* Short-run high-grey-level emphasis, *SRLGE* Short-run low-grey-level emphasis

*Only valid for level *c* compared to level d, all other *p* values being < 0.001

**Only valid for level *a* compared to level *b*, all other *p* values being < 0.001

**Fig. 4:**
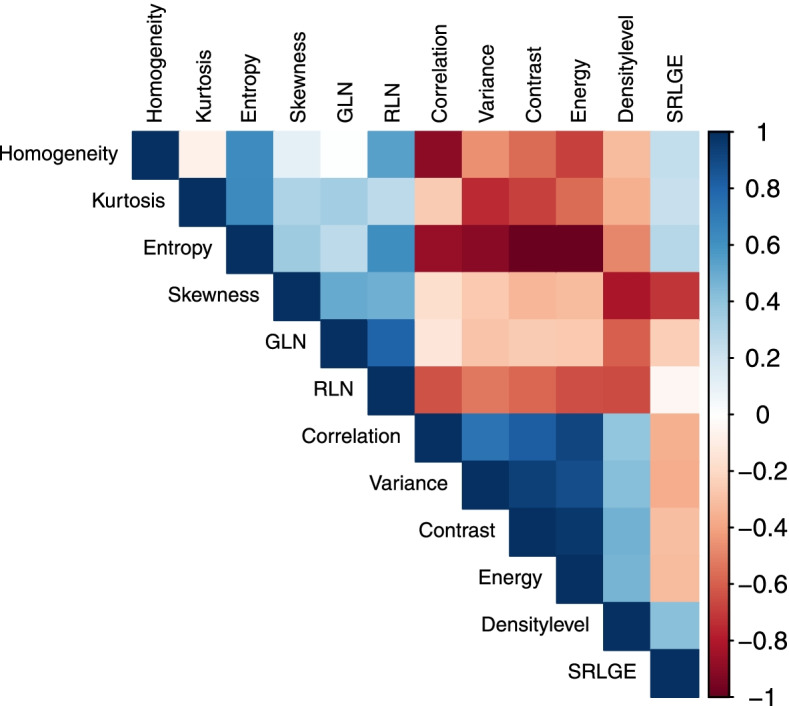
Correlation matrix for the remaining 11 texture features, sorted by hierarchical order. *GLN* Grey-level nonuniformity, *RLN* Run-length nonuniformity, *SRLGE* Short-run low-grey-level emphasis

**Fig. 5:**
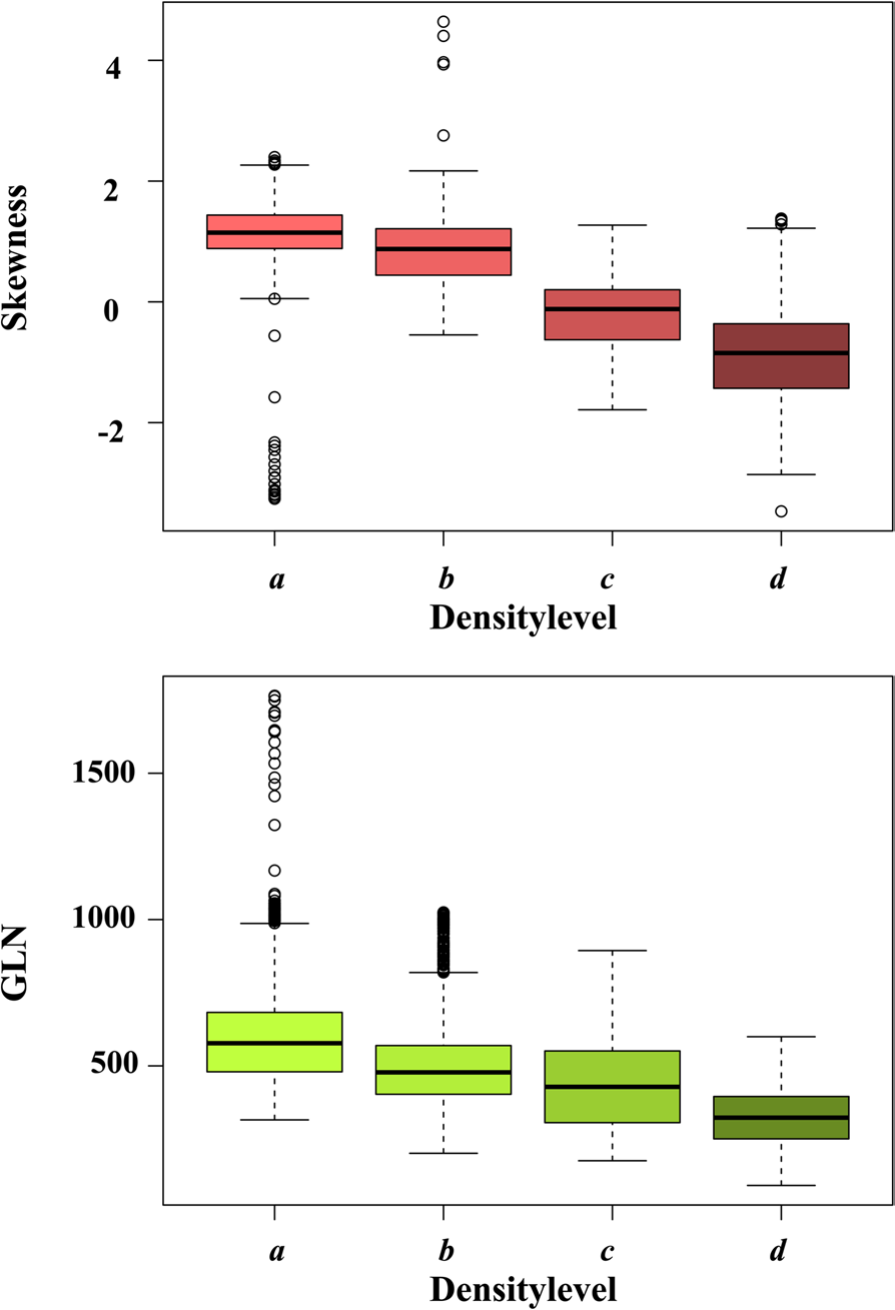
Boxplots of mean values of the two independent texture features derived from texture analysis: skewness and grey-level nonuniformity (GLN)

Of the remaining 11 TFs, 10 correlated significantly with the BD level (Table 3) according to Spearman’s rho. Short-run low grey-level emphasis (SRLGE) was the only feature which showed no significant correlation with the BD level (rho = 0.41, *p* = 0.4). Moreover, SRLGE was independent of most of the other features, but showed high negative correlation with skewness. Skewness, as a first-order feature, showed strong negative correlation with the density level (rho = -0.81, *p* < 0.001), and appeared to be mostly independent of the other features. Cluster analysis exhibited strong correlation within the remaining first-order TFs variance (kurtosis and entropy), without any benefit in distinguishing BD levels (*r* 0.43, -0.35, -0.49, all *p* values < 0.001); neither did contrast, correlation and homogeneity (correlation coefficients 0.48, 0.49, and -0.31; all *p* values < 0.001). GLN, as a second-order feature, showed moderate correlation with skewness and BD (correlation coefficients 0.51 and -0.59, *p* values < 0.001), but appeared to be also mostly independent of the other features. RLN, as the remaining second order feature, revealed moderate negative correlation with BD (correlation coefficient -0.66, *p* < 0.001), but also showed moderate to strong negative correlation with the other first order features (variance, kurtosis, entropy). Moreover, RLN showed strong correlation with GLN (correlation coefficient 0.8, *p* < 0.001), therefore, seemed to be redundant and was further rejected. This left Skewness and GLN as the features that showed a highest independence in the assessment of BD at PC-BCT. Figure 4 graphically summarised the correlation within the TFs in hierarchical order for a better overview. Referring to the mean values, both Skewness and GLN appeared to showed a “linear” decrease with increasing BD (boxplots in Fig. 5). Multinomial logistic regression revealed an overall accuracy on the training dataset of 80.0%. Confusion matrices for the test dataset are listed in Table 4. Highest accuracies were reached for BD levels *a* (86.8%) and *d* (84.5%), whereas predictions for levels *b* (69.0%) and *c* (75.5%) were inferior in our model.

**Table 3:** Spearman’s correlation coefficient and *p* values for each texture feature and breast density level

Texture feature	Spearman correlation coefficient (rho)	***p*** value
Variance	0.43	< 0.001
Skewness	-0.81	< 0.001
Kurtosis	-0.35	< 0.001
Entropy	-0.49	< 0.001
Contrast	0.48	< 0.001
Correlation	0.49	< 0.001
Energy	0.47	< 0.001
Homogeneity	-0.31	0.003
GLN	-0.59	< 0.001
RLN	-0.66	< 0.001
SRLGE	0.41	0.363

*GLN* Grey-level nonuniformity, *RLN* Run-length nonuniformity, *SRLGE* Short-run low-grey-level emphasis

**Table 4:** Confusion matrices for multinomial logistic regression on the test dataset

	Breast density level			
**Predicted**	*a*	*b*	*c*	*d*
*a*	651	98	0	1
*b*	127	517	104	1
*c*	0	103	567	80
*d*	0	4	112	634

### Human readout

Interrater correlation for BD assessment between the radiology resident and the two expert readers was almost perfect (ICC 0.91; 95% confidence interval 0.88−0.94). κ values between the radiology resident and each of the two readers were 0.86 (J.W.) and 0.83 (A.B.) reflecting an almost perfect inter-reader reliability. The overall agreement between the radiology resident and reader 1 was 90% between the resident and reader 2 was 88%. The confusion matrices of the two expert readers, compared to the classification of the radiology resident are shown in Table 5 (*p* values were < 0.001 in each case of statistical comparison). Coefficients of variation for 19 TFs varied from -6.9% to 10.9%. The majority of TFs showed strong reliability with variation coefficients < 5%. Features which showed the strongest correlation with BD also showed the highest coefficients of variance: skewness (-6.93%), GLN (10.9%) and RLN (8.3%). Mean values for each TF and coefficients of variance are listed in Table 6.

**Table 5:** Breast density assessment (^*a–d*^) for 60 representative breast-CT images, performed by two readers (1: J.W.; 2: A.B.), compared to classification given the radiology resident (A.L.)

Radiology resident	Reader 1				Reader 2			
	BD^*a*^	BD^*b*^	BD^*c*^	BD^*d*^	BD^*a*^	BD^*b*^	BD^*c*^	BD^*d*^
BD^*a*^	15	0	0	0	15	0	0	0
	100%	0%	0%	0%	100%	0%	0%	0%
BD^*b*^	0	16	1	0	1	16	0	0
	0%	94%	6%	0%	6%	94%	0%	0%
BD^*c*^	0	2	13	0	0	4	9	2
	0%	13%	87%	0%	0%	27%	60%	13%
BD^*d*^	0	0	3	10	0	0	0	13
	0%	0%	23%	77%	0%	0%	0%	100%

*BD* breast density

^*a*^almost entirely fatty tissue

^*b*^scattered glandular tissue

^*c*^heterogenous dense glandular tissue

^*d*^homogenous dense glandular tissue

**Table 6:** Mean values and coefficients of variance for 19 texture features for the subset of 60 images

Texture feature	Reader 1 (mean)	Reader 2 (mean)	Reader 3 (mean)	Coefficient of variance
Variance	189	186	186	3.23
Skewness	0.653	0.491	0.578	-6.93
Kurtosis	0.412	0.295	0.521	-2.66
Entropy	11.3	11.5	11.6	4.13
Contrast	0.944	0.944	0.942	0.21
Correlation	0.006	0.006	0.006	4.85
Energy	0.443	0.437	0.438	1.11
Homogeneity	5.37	5.41	5.39	0.57
SRE	0.874	0.878	0.877	0.4
LRE	1.82	1.77	1.79	1.91
GLN	1,860	1,550	1,800	10.85
RLN	33,000	28,800	32,200	8.29
RP	0.829	0.835	0.833	0.57
LGRE	0.001	0.001	0.002	5.71
HGRE	1,140	1,150	1,140	0.49
SRLGE	0.001	0.001	0.001	5.6
SRHGE	1,000	1,010	1,000	0.63
LRLGE	0.002	0.003	0.005	6.68
LRHGE	2,300	2,210	2,260	2.76

*GLN* Grey-level nonuniformity, *HGRE* High grey-level run emphasis, *LGRE* Low grey-level run emphasis, *LRE* Long-run emphasis, *LRHGE* Long-run high-grey-level emphasis, *LRLGE* Long-run low-grey-level emphasis, *RLN* Run-length nonuniformity, *RP* Run-percentage, *SRE* Short-run emphasis, *SRHGE* Short-run high-grey-level emphasis, *SRLGE* Short-run low-grey-level emphasis

## Discussion

In our study, we investigated the potential of TA for BD classification in PC-BCT. Except of one feature, the majority of TFs revealed highly significant differences within BD levels. However, correlation coefficients for most TFs revealed only weak correlation with BD levels and were neglected. We were able to derive two TFs, one first-order feature (skewness) and one second-order features (GLN) that demonstrated the strongest correlation with BD density on PC-BCT and were independent of other TFs, therefore, particularly suitable to assess BD in breast-CT.

While BD is known to be an independent risk factor for the development of BC [2], besides the amount of breast glandular parenchyma itself, its distribution is also relevant in BC risk assessment. Following the pioneering work of Wolfe et al. in 1976 [30] numerous studies showed the effect on parenchymal patterns on the individual BC risk [4, 14, 15, 26]. In the common hypothesis, the distribution of fatty, glandular, and stromal breast tissue is related to factors that are associated with the development of BC through unknown biological mechanisms [4]. However, the distribution of the glandular tissue, such as “scattered” and “heterogeneously dense” was not implemented into the BI-RADS classification atlas until the revision in 2013 [5].

While amount and distribution of glandular tissue are more likely associated with the individual BC risk, the transparency of breast tissue is an important factor regarding lesion visibility. Because x-ray absorption of glandular tissue and soft tissue lesions is identical, lesion detectability is highly restricted in dense breasts. Additionally, the effect of tissue-overlay in mammography increases this weak spot. With PC-BCT we are able to observe fatty septae within glandular tissue, which are unique for this new imaging technique. Based on this finding, Wieler proposed a new density atlas for breast density for spiral PC-BCT [25].

Although Tice et al. [31] and Brentnall et al. [32] already demonstrated that considering BD descriptors improves the accuracy of BC risk assessment in the screening population, the discriminative accuracy of many models implementing BD in BC risk estimation remains limited on an individual level [33]. Of note, the standard model for BC risk estimation provided by Gail and Claus is still based on non-modifiable risk factors, such as demographics and genetics [34, 35]. However, the interpretation of BD in mammograms, remains observer-dependent, which might introduce unwanted variability in BC risk assessment. Whereas the relationship between BD and the risk for BC has already been elucidated to a certain extent, the correlation between parenchymal texture features and cancer risk still remains to be clarified [4]. TA has been applied on lesion characterisation or even in the BC risk assessment regarding breast parenchymal distribution with promising results [15]. However, the interrelation between TA data and underlying biological properties has not yet been resolved.

There is large body of literature concerning mammographic texture features for BC detection [15]. Keller et al. [36] compared different prediction models and discovered that TFs outperform common prediction models based on the amount of tissue only. Nevertheless, conventional mammograms are superimposed projection images. Therefore, TFs may reflect skin, subcutaneous fat or overlay effects, *i.e.*, anatomic noise reducing the predictive value of TA in two-dimensional mammograms.

Digital breast tomosynthesis is a pseudo-three-dimensional modality obtained using multiple low-dose two-dimensional x-ray projections with superior tissue visualisation by separating skin and subcutaneous fat from the deeper parenchymal tissue layers. Kantos et al. [4] investigated the association of different texture features with BD in DBT compared to conventional mammograms. They showed that TFs in tomosynthesis have a stronger dependency on the amount of glandular tissue compared to two-dimensional mammogram. Nevertheless, tomosynthesis has low spatial resolution along the *z*-axis and, therefore, remains not completely satisfactory for TA.

Spiral PC-BCT instead, is a truly three-dimensional breast imaging modality suitable for TA. To the best of our concerns, our study is the first investigating the potential of TA in spiral PC-BCT, though there have been investigations on cone-beam CT or positron emission tomography/CT in BC patients [18, 37]. Due to its three-dimensional nature, spiral PC-BCT can provide around 3,500 images (including raw data images and high-resolution images), resulting into a systematically higher evaluation time. To date, the classification for breast density in PC-BCT is not implemented in the BI-RADS classification system and its estimation is highly subjective to the radiologist’s perception. Therefore, there is a urgent need for an objective and observer-independent classification system of BD in PC-BCT.

In recent studies, the implementation of machine learning algorithms using deep convolutional neural networks (CNNs) for the classification of BD in mammography, as well as in spiral PC-BCT, was described [38]. In their latest work, Landsmann et al. [39] reported an accuracy of 85.5% for density assessment in PC-BCT. However, their model appeared to be less linked to the fatty-septae criteria, compared to human decision-making. Typically, in deep learning applications, spatial resolution of images needs to be reduced due to technical constraints regarding hardware memory and calculation time, which is why deep CNNs might be used for BD classification based on the amount and distribution of parenchyma, however, missing the additional feature of the presence of fatty septae in PC-BCT images. Radiomics can detect subtle changes in parenchymal texture, which deep learning might miss. TA therefore, might be able to investigate imaging features linked to fatty septae, which might be substituted by a deep CNN in the training process. For example, skewness and SRLGE showed strong negative correlation with each other, but were mostly independent of all other TF. Moreover, skewness showed the strongest negative correlation with the density level, whereas SRLGE did not show significant correlation with BD at all. This may propose, that skewness and SRLGE show the opposite underlying (unknown) biological difference. In our study we observe similar accuracy (80%) for BD assessment compared to the provided deep learning approach describe by Landsmann et al. [39]. Whereas deep learning requires a large amount of data and high computational power to be trained, TA can also be performed with a small number of images, hence particularly suitable for this new imaging technique. Further studies will have to show whether training a deep learning algorithm with selected TFs might improve the accuracy of BD assessment in PC-BCT.

There are several limitations of this study. First, the single-centre retrospective design including a small number of patients. Second, because of multislice analysis, the same ROI was applied on multiple images, which might introduce some bias in the training data. Third, we only examined the influence of 19 TFs, whereas other studies on TA evaluate more TFs of even higher-orders TFs. However, recent studies on TA in different tissues (*e.g.*, breast, lung, uterus) showed that a lot of the higher-order TFs were not relevant for the differentiation of tissue [17, 40, 41]. Fourth, contrast-enhancement of lesions and the glandular parenchyma using iodinated contrast agents may provide additional information on malignancy and hormonal stimulation of breast tissue. However, the assessment of effects caused by contrast agents was out of the scope of this study.

In conclusion, we were able to show that TA can predict BD with high accuracy. Therefore, TA might be a useful quantitative tool in the classification of BD in spiral PC-BCT, taking into account fatty septae in glandular parenchyma, which are a unique feature of this approach compared to other breast imaging modalities. We also determined two independent TFs that seem particularly suitable to distinguish between different BD levels. These individual features might also be a useful tool in the individual BC risk assessment by reflecting parenchymal composition more precisely.

## Data Availability

The data presented in this study are available on request from the corresponding author. The data are not publicly available due to privacy restrictions.

## Abbreviations

BC: Breast cancer
BD: Breast density
CT: Computed tomography
GLN: Grey-level nonuniformity
ICC: Intraclass correlation coefficient
MRI: Magnetic resonance imaging
PC-BCT: Photon-counting breast CT
RLN: Run-length nonuniformity
ROI: Region of interest
SRLGE: Short-run low-grey-level emphasis
TA: Texture analysis
TF: Texture feature
